# A population pharmacokinetic approach to compare ^51^Cr-EDTA and ^99 m^Tc-DTPA clearances in measuring renal glomerular filtration rate in oncopediatrics

**DOI:** 10.1007/s00467-025-06828-9

**Published:** 2025-05-29

**Authors:** Matthieu Gracia, Victoire Ankaoua, Mathieu Alonso, Marlène Pasquet, Etienne Chatelut

**Affiliations:** 1 Cancer Research Centre of Toulouse (CRCT), Université de Toulouse, Inserm, Toulouse, France; 2 Oncopole Claudius-Regaud, IUCT-Oncopole, 1, Avenue Irène Joliot-Curie, 31059 Toulouse Cedex, France; 3 Unité de Radiopharmacie, CHU de Toulouse, Toulouse, France; 4 Service d’OncoPédiatrie, CHU de Toulouse, Toulouse, France

**Keywords:** ^99 m^Tc-DTPA, ^51^Cr-EDTA, Pediatrics, Glomerular filtration rate, Population pharmacokinetics

## Abstract

**Background:**

^51^Cr-EDTA and ^99 m^Tc-DTPA clearances are two reference methods of determining the glomerular filtration rate. The study aimed to compare these two radioisotopic clearance measurements using an original approach based on population pharmacokinetics in oncopediatrics.

**Methods:**

Plasma concentrations of ^51^Cr-EDTA and ^99 m^Tc-DTPA obtained from, respectively, 40 and 19 children treated with nephrotoxic chemotherapy were simultaneously analyzed while accounting for three covariates (i.e., serum creatinine, plasma cystatin C, body weight) previously described as being related to the pediatric glomerular filtration rate. A binary covariate “GFR measurement” (MES = 0 or 1, respectively, for ^51^Cr-EDTA or ^99 m^Tc-DTPA) was added to the model.

**Results:**

Analysis revealed a non-significant bias (± 95% CI) of − 0.9% ± 11.4% between the two measurements (with ^99 m^Tc-DTPA clearance overestimated).

**Conclusions:**

This result confirmed that both radioisotopic clearances are equivalent in pediatrics, as has been reported in the literature on adults based on intrapatient comparisons. The value of the population approach in comparing the pharmacokinetics of two different compounds is thus demonstrated.

**Graphical Abstract:**

**Figure:**
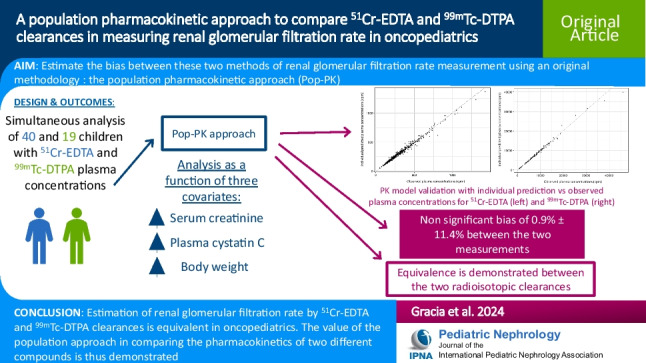
A higher resolution version of the Graphical abstract is available as Supplementary information

**Supplementary Information:**

The online version contains supplementary material available at 10.1007/s00467-025-06828-9.

## Introduction

Chromium-51-ethylenediaminetetraacetate (^51^Cr-EDTA) clearance has been used to measure renal glomerular filtration rate (GFR) for several decades. The production line shutdown of chromium-51 by the European supplier GE Healthcare since 2018, however, has made it necessary to substitute another compound, technetium-99 m-diethylenetriaminepentaacetic acid (^99 m^Tc-DTPA), also known to have glomerular filtration as the sole elimination pathway [1]. It has been shown that ^99 m^Tc-DTPA clearance correlates closely with inulin clearance [2], which is the recognized reference method for GFR measurement. Despite its accuracy, this latter method is difficult to perform, costly, of limited availability and time-consuming, hampering its use in clinical practice [3]. Several studies have compared ^99 m^Tc-DTPA and ^51^Cr-EDTA clearance in adults. They concluded that the difference between both measurement methods is minimal [4–11]. However, no similar study has been performed in children. Grönroos et al. did highlight a correlation between cystatin C and creatinine concentrations with ^99 m^Tc-DTPA and ^51^Cr-EDTA clearances in 36 children but they did not quantify the difference between both measurements [12].

The objective of our study was to quantify the bias between ^99 m^Tc-DTPA and ^51^Cr-EDTA clearances in pediatrics using an original approach based on population pharmacokinetic (PK) modelling. This methodology consists of simultaneously analyzing plasma concentrations vs. time for a compound (usually a drug; a radioisotope in our study) in a group of patients. It is thus possible to quantify interindividual PK variability and evaluate the relationship between demographic, morphological, and biological variables, and PK parameters such as elimination clearance (CL). We used this approach previously in pediatrics to analyze ^51^Cr-EDTA plasma concentrations vs. time to obtain an equation estimating GFR from three covariates (i.e., serum creatinine or Scr, plasma cystatin C or PcysC, body weight or BW) [13]. We then evaluated this equation (“CYSPED”) prospectively using ^99 m^Tc-DTPA obtained from 19 children. We were able to confirm that this equation was more accurate than those previously proposed, for example by Schwartz et al. based on Scr and body weight covariates with [14] or without [15] cystatin C [16]. These previous analyses, as all those usually based on the population PK approach, consisted in analyzing data of a specific compound (a drug or, for our previous works, either ^51^Cr-EDTA or ^99 m^Tc-DTPA). The originality of the current analysis is to use the population PK approach for simultaneously analyzing data of two different compounds (i.e., ^51^Cr-EDTA or ^99 m^Tc-DTPA corresponding to the “Cysped” clinical trial [13] and “CysPedVal” [16]) with the aim of quantifying the bias between both radioisotopic measurement methods.

## Methods

### Patients and data

The data (i.e., radioisotopic plasma concentrations vs. time) were those obtained from 40 children included in the Cysped clinical trial (NCT02822404) for ^51^Cr-EDTA clearance between February 2012 and September 2015 [13] and those from 19 children in the CysPedVal analyze (RnIPH 2020101) for ^99 m^Tc-DTPA clearance between December 2020 and June 2022 [16]. Inclusion criteria were patients between 0 and 18 years of age, with a body weight over 4 kg, and treated with cisplatin and/or ifosfamide requiring GFR monitoring to evaluate the nephrotoxicity of chemotherapy. Informed signed consent was obtained from parents or children’s legal representatives. Key patient characteristics are shown in Table 1. Creatinine serum levels were determined by an enzymatic method using creatininase with the Cobas 8000 analyzer (Roche France, Meylan). Cystatin C plasma levels were measured by an automated particle-enhanced nephelometric immunoassay (PENIA). Data were collected at baseline (before chemotherapy) and at the end of treatment. ^51^Cr-EDTA and ^99 m^Tc-DTPA were administered intravenously as bolus doses twice for each patient (before chemotherapy and at the end of treatment), and four blood samples were taken from the opposite arm at 90-, 110-, 130-, and 150-min post-injection. The radionuclide dose was 2.072 MBq for ^51^Cr-EDTA and 6, 9, or 12 MBq for ^99 m^Tc-DTPA depending on patient weight (respectively, < 10 kg, 10–20 kg, or > 20 kg). Between one and five (median three) GFR measurements were taken per patient depending on the number of cycles of chemotherapy administered. No patient successively received both radioisotopic tracers.

**Table 1:** Children’s characteristics at baseline (before the first glomerular filtration rate measurement) and their type of malignancy

Characteristics	EDTA group mean (range) *n* = 40	DTPA group mean (range) *n* = 19	*P*-value
Age (years)	10.6 (1.4–17.8)	10.1 (2.0–16.3)	0.242
Gender (male/female)	18/22	8/11	0.570
Body weight (kg)	34.3 (9.07–72.0)	42.9 (10.0–91.8)	1.10^–4^
Body height (cm)	138 (48–186)	141 (86–179)	0.381
Body surface area	1.13 (0.37–1.89)	1.27 (0.49–2.16)	0.002
Serum creatinine (µmol/L)	42 (11–102)	42 (8–76)	0.996
Plasma cystatin C (mg/L)	0.76 (0.26–1.36)	0.82 (0.56–1.14)	8.10^–6^
Type of malignancy (number)			
Osteosarcoma	21	12	
Neuroblastoma	6	3	
Germinal tumor	4	3	
Rhabdomyosarcoma	3	0	
Others	6	1	

### Pharmacokinetic analysis

The data were analyzed using NONMEM software version 7.4.3, and the PIRANA program version 21.11.1 using a monocompartmental model based on two PK parameters: elimination clearance (CL) and distribution volume (V). A first-order conditional estimation with interaction (FOCEI) method and a proportional error model for residual and interpatient variabilities were used. Interoccasion variability of CL was included. The typical value for V was expressed as being proportional to body weight (BW, in kg): TVV = θ.BW. The starting point of the PK analysis was the population model previously identified; the typical value of CL was expressed according to the CYSPED equation: TVCL = θ_1_.(Scr/42.25)^θ2^.(PcysC/0.76)^θ3^.(BW/34.3)^θ4^, with Scr for serum creatinine (in µmol/L), and PcysC for plasma cystatin C levels (in mg/L). This model was evaluated using backward elimination (removal of each covariate separately). Only those covariates associated with an increase in objective function value (OFV) of more than 6.635 (*p* < 0.01) were retained in the final model. Once this model was validated, the bias between the two radioisotopic tracers was determined by adding the term (1 – θ.MES), where MES = 0 or = 1, respectively, for ^51^Cr-EDTA or ^99 m^Tc-DTPA, to the final covariate model for TVCL and TVV. Considering that some observed plasma concentrations corresponded to ^51^Cr-EDTA data and others to ^99 m^Tc-DTPA data, specific residual variability was allocated to ^51^Cr-EDTA and ^99 m^Tc-DTPA data.

## Results

**Fig. 1:**
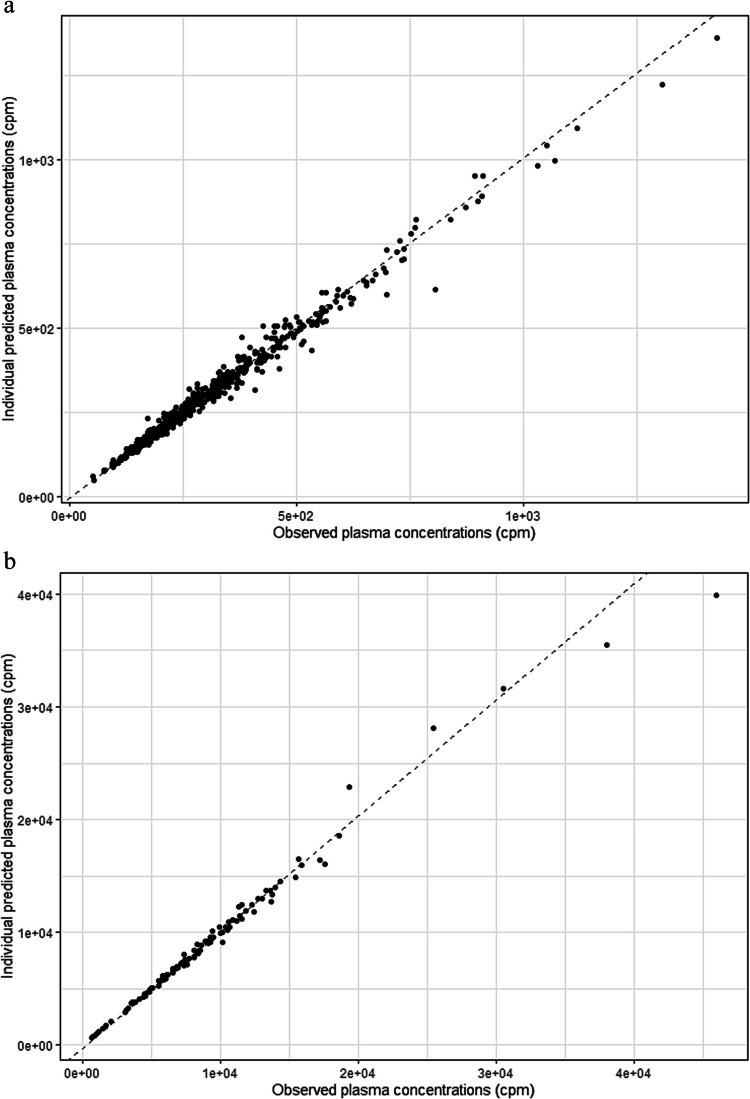
Individual predicted vs. observed plasma concentrations for ^51^Cr-EDTA (Fig. 1a) and for.^99 m^tc-DTPA (Fig. 1b)

The PK of radioisotopic tracers was adequately described by a monocompartmental model as shown by the small values of residual variability (Table 2) and Fig. 1 presenting individual predicted vs. observed ^51^Cr-EDTA and ^99 m^Tc-DTPA plasma concentrations. Deletion of each covariate from the initial model based on the three covariates was associated with a significant increase in OFV. This final model had only slightly changed coefficient values compared with the CYSPED equation. The value (± 95%CI) of the coefficient, corresponding to the MES covariate added to the final model, was − 0.009 (± 0.114) for CL and + 0.017 (± 0.143) for V. The bias between the clearances of ^51^Cr-EDTA and ^99 m^Tc-DTPA through MES covariate parameter is very small (and not significantly different to 0) reflecting equivalence between both investigating clearances techniques. To evaluate the benefit of considering patient characteristics in quantifying the bias between radioisotopic clearances, values of the MES covariate were also determined using alternative models (i.e., a model with no covariate or only one or two covariates among Scr, PcysC, and BW). These results are shown in Table 3 together with corresponding interindividual variability values (i.e., interindividual variability not explained by the covariates considered in the model).

**Table 2:** Parameters of the final pharmacokinetic model for ^51^Cr-EDTA and ^99 m^Tc-DTPA radioisotopic tracers

Pharmacokinetic parameters^¤^	Mean ± 95% CI
Clearance (CL) (mL/min): typical value of CL = θ1.(Scr/42.25)^θ3^.(PcysC/0.7738)^θ4^.(BW/35.70) ^θ5^.(1—θ6.MES)	θ1 = 80.8 ± 3.1 θ3 = − 0.568 ± 0.156 θ4 = − 0.295 ± 0.168 θ5 = + 1.06 ± 0.145 θ6 = − 0.009 ± 0.114
Interoccasion variability (CV%)	14.5%
Interindividual variability (CV%)	11%
Volume of distribution (V) (mL): typical value of V = θ7.BW.(1—θ8.MES)	θ7 = 256 ± 20 θ8 = + 0.017 ± 0.143
Interindividual variability (CV%)	20.9%
Residual errors (CV%)	
^51^Cr-EDTA plasma concentrations	4.99%
^99 m^Tc-DTPA plasma concentrations	7.18%

*Scr* serum creatinine, *PcysC* plasma cystatin C, *BW* body weight, MES = 0 or = 1, respectively, for ^51^Cr-EDTA or ^99 m^Tc-DTPA data

**Table 3:** Comparison of bias and interindividual variability (IIV) of radioisotopic clearance estimated according to the covariate pharmacokinetic model

Covariate model	Bias ± 95% confidence interval	% Coefficient of variation (IIV)
Final based on Scr, PcysC, and BW	− 0.009 ± 0.11	11.0
Based on Scr, and BW	− 0.035 ± 0.10	10.5
Based on PcysC, and BW	− 0.119 ± 0.15	13.3
Based on Scr, and PcysC	− 0.280 ± 0.38	45.8
Based on BW	− 0.053 ± 0.17	15.1
Based on Scr	− 0.217 ± 0.39	51.7
Based on PcysC	− 0.288 ± 0.35	43.0
Without covariate	− 0.215 ± 0.32	41.4

*Scr* serum creatinine, *PcysC* plasma cystatin C, *BW* body weight

## Discussion

The bias we obtained between the clearances of ^51^Cr-EDTA and ^99 m^Tc-DTPA in 59 children is very small and is both accurate (i.e., − 0.9% ± 11%) and consistent with values previously observed in adults. Biggi et al. observed in 54 patients a greater clearance for ^99 m^Tc-DTPA than for ^51^Cr-EDTA by 2.9% [8]. In 30 patients, Fleming et al. observed a 7.6% “systematically higher” value for ^99 m^Tc-DTPA [9]. McMeekin et al. observed a bias of 2.9% with ^99 m^Tc-DTPA giving the higher result when the three-sample slope-intercept GFR was used [10]. However, there was no statistically significant difference between the two radioisotopic tracers when GFR was calculated by a full characterization of the plasma clearance curve. All of these results in adults were obtained by simultaneously administrating both compounds. This methodology is the gold standard in comparing the two radioisotopic clearances since inter-volunteer and interoccasion variability of GFR do not impact results. This question has not yet been explored in children, most likely for ethical considerations limiting the administration of two radioisotopic tracers merely for confirmatory evaluation. In a sample of 29 children (aged 1 month to 12 years), Gutte et al. compared non-invasive ^99 m^Tc-DTPA renography and single-sample ^51^Cr-EDTA clearance concluding that they were comparable with an average difference (^99 m^Tc-DTPA renography – ^51^Cr-EDTA GFR) of 3 mL/min [17].

The second main aim of our study (in addition to quantifying the bias between the two radioisotopic clearances) is to demonstrate the benefit of the population approach allowing us to simultaneously analyze ^51^Cr-EDTA and ^99 m^Tc-DTPA data. Given that individual GFR can be accurately estimated by using morphological (body weight) and biological (serum creatinine and/or cystatin C plasma levels) characteristics, considering these covariates at every cycle eliminates interindividual and interoccasion variabilities. This is demonstrated by comparing the different values of bias obtained depending on the covariate model used (Table 3). The lowest bias corresponds to the final covariate model; covariate models without body weight are associated with higher bias and less precision. The three models including body weight (alone or with one of the creatinine or cystatin C covariates) resulted in intermediate values. Overall, the more fully patients are described, the less bias between the two radioisotopic clearances and the greater the precision of this value. Moreover, the additional benefit of this methodology is to make it possible to simultaneously analyze subgroups of patients which differ with regard to certain variables. Indeed, the children with ^99 m^Tc-DTPA clearance had significantly higher body weights and higher concentrations of plasma cystatin C on average (Table 1) than those with ^51^Cr-EDTA clearance. But the fact that both these characteristics are accounted for in the CYSPED equation ensures that both subgroups can be considered comparable in the analysis. Furthermore, it should be noted that the benefit of considering plasma cystatin C to evaluate the renal function of children has been confirmed by several other recent publications [18, 19].

Beyond the clinical interest of our study (i.e., ^51^Cr-EDTA and ^99 m^Tc-DTPA clearances may be interpreted similarly in pediatrics), our research supports the use of the population approach to analyze radioisotopic plasma concentrations vs. time. This analysis confirmed that adding ^99 m^Tc-DTPA data from 19 children to ^51^Cr-EDTA data from 40 children does not significantly change the CYSPED equation: GFR = 77.5.(Scr/42.25)^−0.423^.(PcysC/0.76)^−0.267^.(BW/34.3)^+0.934^ (40 patients) became GFR = 80.8.(Scr/42.25)^−0.568^.(PcysC/0.76)^−0.295^.(BW/34.3)^+1.06^ (59 patients). These results show the value of this equation in estimating GFR in children when radioisotopic clearance is difficult to assess, for example, when repetitive measures are required for monitoring nephrotoxicity during chemotherapy corresponding to standard treatment, or even to new anticancer compounds being developed. However, radioisotopic clearance remains indicated in all clinical situations requiring accurate GFR determination such as for pediatric patients with chronic renal disease and adult kidney graft donors.

## Conclusion

Our study was based on a population pharmacokinetic approach to evaluate the bias between ^51^Cr-EDTA and ^99 m^Tc-DTPA clearances in oncopediatrics. We conclude that the glomerular filtration rate estimation is equivalent with both, regardless of the radioisotopic tracer used.

## Supplementary Information

Below is the link to the electronic supplementary material.

Graphical Abstract (PPTX 129 KB)

Supplementary file2 (DOCX 14 KB)

## Data Availability

Data are available on request from the corresponding author.
