# Supplementary material for: A population pharmacokinetic approach to compare 51Cr-EDTA and 99 mTc-DTPA clearances in measuring renal glomerular filtration rate in oncopediatrics
Source: Pediatr Nephrol. 2025 May 29;40(10):3163–8. doi: 10.1007/s00467-025-06828-9 (PMC12401762; doi:10.1007/s00467-025-06828-9)
Supplement: Supplementary file 1 — Graphical Abstract (PPTX 129 KB) [file 467_2025_6828_MOESM1_ESM.pptx]

## Slide 1
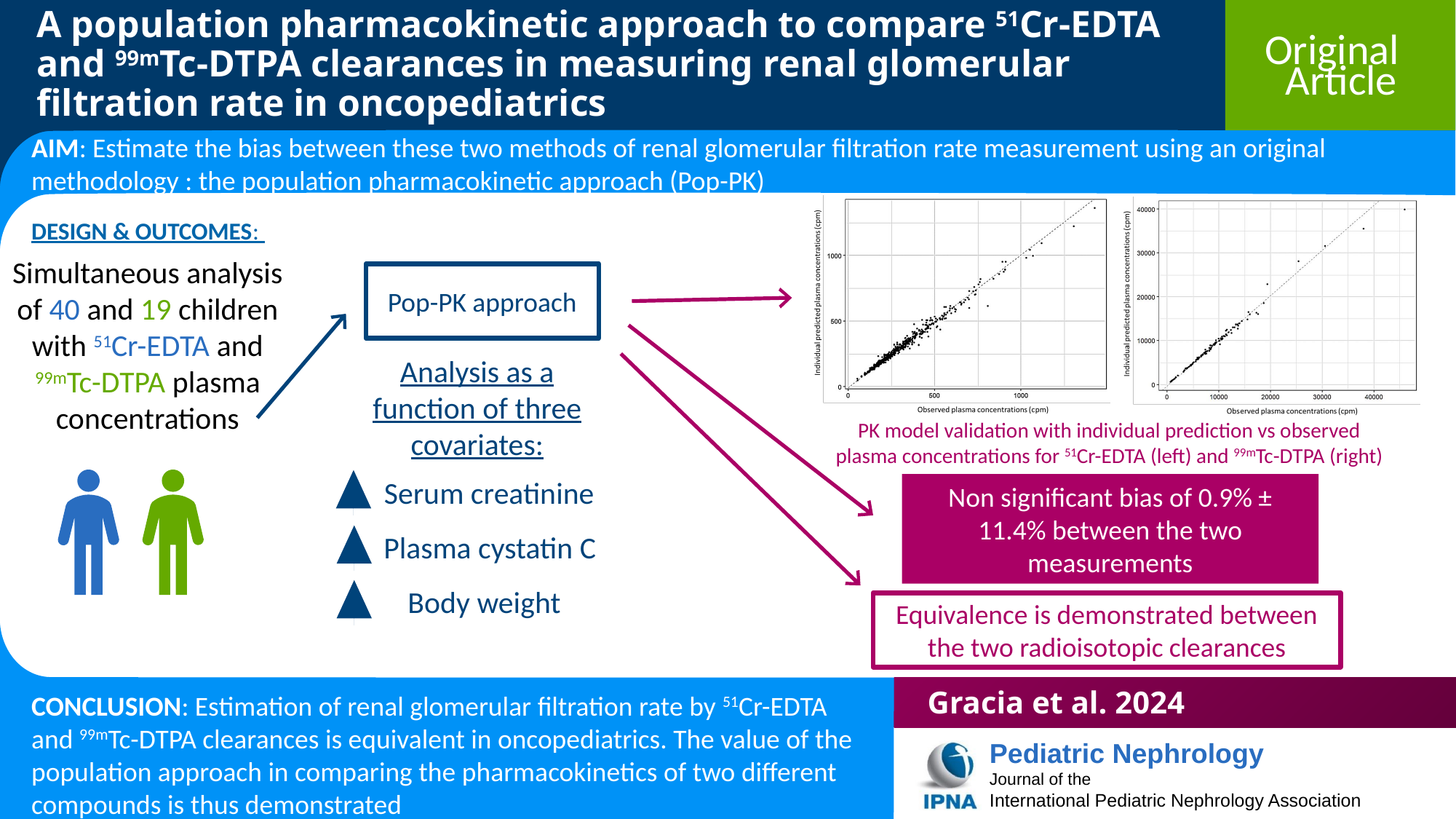

A population pharmacokinetic approach to compare 51Cr-EDTA and 99mTc-DTPA clearances in measuring renal glomerular filtration rate in oncopediatrics
AIM: Estimate the bias between these two methods of renal glomerular filtration rate measurement using an original methodology : the population pharmacokinetic approach (Pop-PK)
DESIGN & OUTCOMES:
Simultaneous analysis of 40 and 19 children with 51Cr-EDTA and 99mTc-DTPA plasma concentrations
Pop-PK approach
Analysis as a function of three covariates:
PK model validation with individual prediction vs observed plasma concentrations for 51Cr-EDTA (left) and 99mTc-DTPA (right)
Serum creatinine
Non significant bias of 0.9% ± 11.4% between the two measurements
Plasma cystatin C
Body weight
Equivalence is demonstrated between the two radioisotopic clearances
Gracia et al. 2024
CONCLUSION: Estimation of renal glomerular filtration rate by 51Cr-EDTA and 99mTc-DTPA clearances is equivalent in oncopediatrics. The value of the population approach in comparing the pharmacokinetics of two different compounds is thus demonstrated
