# Supplementary material for: A population pharmacokinetic approach to compare 51Cr-EDTA and 99 mTc-DTPA clearances in measuring renal glomerular filtration rate in oncopediatrics
Source: Pediatr Nephrol. 2025 May 29;40(10):3163–8. doi: 10.1007/s00467-025-06828-9 (PMC12401762; doi:10.1007/s00467-025-06828-9)
Supplement: Supplementary file 2 — Supplementary file2 (DOCX 14 KB) [file 467_2025_6828_MOESM2_ESM.docx]

Supplementary materials

The data analysed in the current manuscript were collected in two steps. A first clinical trial sought to determine the best markers of renal toxicity in anticancer treatment registered in ClinicalTrials.gov as NCT0282240 [1]: <https://clinicaltrials.gov/study/NCT02822404?term=cysped&viewType=Card&rank=1>.

This was followed by a study based on the use of samples leftover from patient care to prospectively evaluate the previously determined equation (registered as RnIPH 2020-101 at the Toulouse University Hospital in compliance with the MR-004 reference methodology of the French Data Protection Agency) [2]. Informed consent was obtained before the first cycle of chemotherapy.

1. Lambert M, White‐Koning M, Alonso M et al (2021) Plasma cystatin C is a marker of renal glomerular injury in children treated with cisplatin or ifosfamide. Pediatr Blood Cancer 68:e28747. https://doi.org/10.1002/pbc.28747

2. Lambert M, Alonso M, Munzer C et al (2024) Prospective validation of an equation based on plasma cystatin C for monitoring the glomerular filtration rate in children treated with cisplatin or ifosfamide for cancer. Cancer Chemother Pharmacol 93:393–395. https://doi.org/10.1007/s00280-023-04597-6
